# Maximum likelihood estimation of a social relations structural equation model

**DOI:** 10.1007/s11336-020-09728-z

**Published:** 2020-10-22

**Authors:** Steffen Nestler, Oliver Lüdtke, Alexander Robitzsch

**Affiliations:** 1 Institut für Psychologie, University of Münster, Fliednerstr. 21, 48149 Münster, Germany; 2 Leibniz Institute for Science and Mathematics Education, Kiel, Germany; 3 Centre for International Student Assessment, Kiel, Germany

**Keywords:** social relations model, structural equation modeling, maximum likelihood estimation

## Abstract

The social relations model (SRM) is widely used in psychology to investigate the components that underlie interpersonal perceptions, behaviors, and judgments. SRM researchers are often interested in investigating the multivariate relations between SRM effects. However, at present, it is not possible to investigate such relations without relying on a two-step approach that depends on potentially unreliable estimates of the true SRM effects. Here, we introduce a way to combine the SRM with the structural equation modeling (SEM) framework and show how the parameters of our combination can be estimated with a maximum likelihood (ML) approach. We illustrate the model with an example from personality psychology. We also investigate the statistical properties of the model in a small simulation study showing that our approach performs well in most simulation conditions. An R package (called srm) is available implementing the proposed methods.

The social relations model (SRM) is a conceptual and mathematical approach that can be applied to disentangle the components of interpersonal judgments and behaviors. It is used across different psychological disciplines (see Kenny et al. 2006), including personality and social psychology, educational psychology, clinical psychology, and organizational psychology (see the SRM bibliography available at http://davidakenny.net/doc/srmbiblio.pdf). For example, social and personality psychologists use the SRM to better understand the liking between unacquainted individuals (e.g., Küfner et al. 2012; Leckelt et al. 2015; Salazar-Kämpf et al. 2018). Educational psychologists use the model to examine students’ performance in learning groups and to determine which students profit the most from such groups (e.g., Horn et al. 1998). Finally, the SRM is used in clinical psychology to better understand the perceptions that occur during group psychotherapy (e.g., Christensen and Feeney 2016).

Typically, the SRM is applied to a *single* round-robin variable. However, it is common practice to measure not just one but several round-robin variables that pertain to a certain construct. In the social psychology example, researchers may have assessed liking by using three round-robin variables: ’I like this person,’ ’I would like to get to know this person,’ and ’I would like to become friends with this person’ (see Salazar-Kämpf et al. 2018). Researchers might also have assessed multiple constructs with multiple round-robin variables. In our example, not only might researchers have measured liking with different liking indicators but perhaps also extraversion perceptions with different round-robin indicators.

Although the univariate social relations model has been extended to capture multivariate round-robin data as well (Card et al. 2008; Kenny 1994; Nestler 2018), such approaches are limited because they were carved out to estimate a saturated model (i.e., all covariances between the round-robin variables are estimated). Hence, they can*not* be applied to examine structural hypotheses concerning the multivariate relations between SRM effects measured by different round-robin variables. In our example, the researcher may be interested in examining whether the three liking variables are equally good measures of a latent perceiver-effect factor or a latent target-effect factor, respectively, or he or she may want to test whether a latent target effect for liking can be used to predict a latent target effect for extraversion.

To investigate such questions, researchers often use a two-step approach in which the SRM effects are estimated for each round-robin variable in the first step (e.g., the target effects are estimated for each liking indicator, see Kenny et al. 2006). In the second step, the estimates are then used in statistical models such as confirmatory factor analysis (CFA) models or structural equation models (SEM, see Bollen 1989; Brown 2006; Kline 2016, for introductions). However, this two-step approach is problematic because the estimated SRM effects might not be reliable estimates of the true SRM effects, thus resulting in distorted estimates of the multivariate structural relations (Lüdtke et al. 2018; Nestler et al. 2015).

A far better approach would be to apply a one-step approach, that is, to estimate the parameters in an SRM confirmatory factor analysis or in an SRM structural equation model. However, this would require the SRM to be combined with the SEM framework. The purpose of the present article is to introduce such a combination and to show how the parameters of this social relations-structural equation model (SR-SEM) can be estimated with a maximum likelihood (ML) approach. In the following, we begin by briefly describing the SRM for a single round-robin variable. Thereafter, we introduce a framework that allows us to combine the SRM with the SEM framework. We then derive the ML approach to estimate the model’s parameters. Finally, we illustrate the model using a real-world example, and we present a simulation study in which we investigate the performance of the ML approach when applied to a social relations model-confirmatory factor analysis (SR-CFA).

## Social Relations Model

The SRM is typically used to analyze data stemming from the round-robin design. In this design, every member of a group is asked to judge every other member of the group and is also judged by every group member with respect to a certain variable. For instance, group members may be asked to indicate how much they like each other group member. According to the univariate SRM (see Kenny 1994; Kenny et al. 2006, for an overview), person *i*’s judgment of person *j* consists of four components:

1 $$\begin{aligned} y_{ij} = \beta + p_{i} + t_{j} + r_{ij}. \end{aligned}$$

Here, $\beta$ is the grand mean of the round-robin variable across all judgments. $p_{i}$ is the perceiver effect of individual *i*, and $t_{j}$ is the target effect of individual *j*. Both effects are person-level effects: The perceiver effect describes the extent to which *i* tends to judge others in a certain way in general. In terms of our example, it reflects how much *i* likes others on average. The target effect is the extent to which person *j* tends to be judged in a certain way in general. For example, how much *j* is liked by all other group members. Finally, $r_{ij}$ denotes the relationship effect. These effects are located at the dyad level and describe the unique component of the judgment after the individual-level effects have been removed. In our example, the relationship effect denotes *i*’s unique liking of *j*.

The distinctiveness of the SRM is best illustrated when we write the two judgments of a specific dyad *d* as a bivariate outcome vector:

2 $$\begin{aligned} \varvec{y}_d = \begin{pmatrix} y_{ij} \\ y_{ji} \end{pmatrix} = \begin{pmatrix} 1 \\ 1 \end{pmatrix} \beta + \begin{pmatrix} 1 &{}\quad 0 \\ 0 &{} \quad 1 \end{pmatrix} \begin{pmatrix} p_{i} \\ t_{i} \end{pmatrix} + \begin{pmatrix} 0 &{}\quad 1 \\ 1 &{} \quad 0 \end{pmatrix} \begin{pmatrix} p_{j} \\ t_{j} \end{pmatrix} + \begin{pmatrix} 1 &{}\quad 0 \\ 0 &{} \quad 1 \end{pmatrix} \begin{pmatrix} r_{ij} \\ r_{ji} \end{pmatrix} \end{aligned}$$

where $y_{ij}$ is the aforementioned judgment of *i* concerning *j*, and $y_{ji}$ is *j*’s judgment of *i*. As Eq. 2 shows, the central feature of the SRM is that the round-robin judgments contain information about the perceiver effect and the target effect of person *i* as well as person *j* and the opposing relationship effects of a specific dyad. This is important insofar as it allows the variances of the person-level and dyad-level effects to be estimated as well as the respective covariance. Specifically, a bivariate normal distribution is assumed for the perceiver effect and the target effect of person *i*

3 $$\begin{aligned} \begin{pmatrix} p_{i} \\ t_{i} \end{pmatrix} \sim MVN(0, \varvec{{{\varSigma }}}_p) \text { with } \varvec{{{\varSigma }}}_p = \begin{pmatrix} \sigma ^2_{p} &{} \quad \sigma _{pt} \\ &{} \quad \sigma ^2_{t} \end{pmatrix} \end{aligned}$$

Here, $\sigma ^2_{p}$ denotes the variance of the perceiver effects. In our example, the perceiver variance measures how much perceivers differ in their average liking judgments. $\sigma ^2_{t}$ is the variance of the target effects describing, for example, whether some targets are liked more on average than other targets. Finally, $\sigma _{pt}$ is the covariance between the two effects, and it measures the extent to which the two person-level effects are associated. In our example, a positive covariance would indicate that individuals who like others on average are also liked more on average. The relationship effects are also assumed to follow a bivariate normal distribution

4 $$\begin{aligned} \begin{pmatrix} r_{ij} \\ r_{ji} \end{pmatrix} \sim MVN(0, \varvec{{{\varSigma }}}_r) \text { with } \varvec{{{\varSigma }}}_r = \begin{pmatrix} \sigma ^2_{r} &{} \quad \sigma _{rr} \\ &{} \quad \sigma ^2_{r} \end{pmatrix} \end{aligned}$$

where the relationship variance, $\sigma ^2_{r}$, measures the extent to which dyads differ in their unique perceptions. In our example, the relationship variance describes whether dyads differ in their unique liking perceptions. The relationship covariance, $\sigma _{rr}$, describes the extent to which the relationship effects are reciprocated. A positive covariance in the example would indicate that *i*’s liking of *j* goes along with *j*’s liking of *i*.

We can use Eq. 2 together with Eqs. 3 and 4 to derive the expectation and the covariance matrix of the two judgments of dyad *d*:

5 $$\begin{aligned} \varvec{\mu }_{\varvec{y}_d}&= \begin{pmatrix} 1 \\ 1 \end{pmatrix} \beta \nonumber \\ \varvec{{{\varSigma }}}_{\varvec{y}_d}&= \begin{pmatrix} \sigma ^2_{p} + \sigma ^2_{t} &{} \quad 2\sigma _{pt} \\ 2\sigma _{pt} &{} \quad \sigma ^2_{p} + \sigma ^2_{t} \end{pmatrix} + \begin{pmatrix} \sigma ^2_{r} &{} \quad \sigma _{rr} \\ \sigma _{rr} &{} \quad \sigma ^2_{r} \end{pmatrix} \end{aligned}$$

where $\varvec{\mu }_{\varvec{y}_d}$ denotes the expectation and $\varvec{{{\varSigma }}}_{\varvec{y}_d}$ is the covariance matrix. Equation 5 shows that the expectation of a single round-robin judgment $y_{ij}$ is $\mu _{y_{ij}}$ = $\beta$ and that the variance is $\sigma ^{2}_{y_{ij}} = \sigma ^2_{p} + \sigma ^2_{t} + \sigma ^2_{r}$.

Most researchers use an analysis of variance (ANOVA) approach to estimate the variance and covariance parameters of the SRM (Bond and Lashley 1996; Lashley and Bond 1997; Warner et al. 1979). However, there are a number of problems (see e.g., Lüdtke et al. 2013; Nestler 2016; Snijders and Kenny 1999) associated with this type of method-of-moment estimator as it is implemented in current software (e.g., the R package TripleR or the Fortran-based SOREMO, Schönbrodt et al. 2017; Kenny 1998). For example, it is difficult to obtain standard errors for the SRM parameters when three or more round-robin variables have been assessed (e.g., Gill and Swartz 2001). It is also difficult to extend the approach to models for longitudinal round-robin data (Nestler et al. 2017). Furthermore, it is impossible to include the effects of covariates on person-level or dyad-level effects (Lüdtke et al. 2018). As a result of this, alternative approaches such as ML (Li and Loken 2002; Li 2006; Nestler 2016, 2018, Snijders and Kenny 1999) or Bayesian estimation (Gill and Swartz 2001; Hoff 2005; Lüdtke et al. 2013, 2018) have been proposed. An advantage of these last two approaches is that they can be carved out for more complex data situations such as when multiple round-robin variables have been measured. Here, we use an ML approach to estimate the parameters of a combination of the SRM with SEMs.

Before we introduce this combination, it is important to note that round-robin data are typically assessed in a multiple round-robin group design that allows researchers to investigate whether the average level of ratings varies across groups. However, differences between groups are usually not of great interest to researchers. Therefore, we are not considering these effects in our expositions and assume that they have been removed from the model by applying, for example, a fixed effect approach (Lüdtke et al. 2013) or group-mean centering (Nestler 2016).

## A Combination of the SRM with Structural Equation Models

Very often, researchers have measured not just a single round-robin variable but rather multiple round-robin variables. In this case, they could use the multivariate SRM (e.g., Card et al. 2008; Nestler 2018) that allows them to compute the SRM variance and covariance parameters for each single round-robin variable. Cross-variable covariance parameters can also be obtained that result from relating SRM effects of the different round-robin variables. Alternatively, they could use a variant of the multivariate SRM that was introduced by Kenny and Livi (2009) and that differentiates between stable and unstable SRM effects. As a CFA, this would translate into a model in which the different round-robin indicators would load in the latent factor with a loading of 1 (e.g., a latent perceiver effect factor) and in which the error variance terms of the indicators are constrained to be of the same value. A disadvantage of these models is that they could *not* be used to test structural hypotheses concerning multivariate relations between SRM effects or only very limited ones (i.e., a single-factor model with equal factor loadings). Hence, researchers could *not* investigate whether three liking variables are equally good indicators of a latent perceiver liking factor and/or of a latent target liking factor. Furthermore, they could not examine whether the target effect of one round-robin variable can be used to predict the perceiver effect of another round-robin variable. To examine such questions—without relying on a statistically problematic two-step approach—requires a combination of the SRM with the confirmatory factor analysis (CFA) model or the structural equation model (SEM).

To combine the SRM with the SEM, we assume that data from $i = 1, \ldots , I$ persons nested in $d = 1, \ldots , D$ dyads are available. Furthermore, we assume that $k = 1,\ldots , K$ round-robin variables have been assessed. In the following, we first describe the basic equations of the SR-SEM. Thereafter, we derive the mean structure and the covariance structure of the multivariate round-robin data vector given our model definition. This is followed by the derivation of an ML estimator for the SR-SEM parameters.

### Basic Equations

Our combination of the SRM with the SEM is based on writing the vector of *all* round-robin judgments $\varvec{y}$ in terms of the person effects for each individual *i* and the relationship effects for each dyad *d*

6 $$\begin{aligned} \varvec{y} = \varvec{X} \varvec{\beta } + \sum _{i=1}^I \varvec{Z}_i \varvec{u}_i + \sum _{d=1}^D \varvec{W}_d \varvec{r}_d , \end{aligned}$$

where $\varvec{X}$ is a design matrix that relates the entries in $\varvec{\beta }$ to the entries in $\varvec{y}$. In most cases, $\varvec{\beta }$ will contain the means of the *K* round-robin variables. Then, $\varvec{X}$ contains only 0s and 1s (see below for an example). However, when one wants to consider the round-robin group effects by applying a fixed effect approach (Lüdtke et al. 2013) or when one wants to model a linear time trend in case of longitudinal round robin data (Nestler et al. 2020), then $\varvec{\beta }$ and $\varvec{X}$ would look differently. $\varvec{Z}_i$ and $\varvec{W}_d$ are also design matrices. They relate the entries in $\varvec{y}$ to the person effects of individual *i* contained in $\varvec{u}_i$ and the relationship effects of dyad *d* in $\varvec{r}_d$, respectively. They always contain 0s and 1s.^1^

For an illustration, we take one dyad (*i*, *j*) out of our larger data set and assume that three round-robin variables have been assessed (see Appendix A for another illustrative example of the basic equation). Equation 6 is then a concatenation of the *K* bivariate round-robin judgments (see Eq. 2 where *K* = 1). In this case, Eq. 6 is

7 $$\begin{aligned} \varvec{y}&= \varvec{X} \varvec{\beta } + \varvec{Z}_1 \varvec{u}_1 + \varvec{Z}_2 \varvec{u}_2 + \varvec{W}_1 \varvec{r}_1 \Leftrightarrow \nonumber \\ \begin{pmatrix} y_{ij1} \\ y_{ji1} \\ y_{ij2} \\ y_{ji2} \\ y_{ij3} \\ y_{ji3} \end{pmatrix}&= \begin{pmatrix} 1 &{} \quad 0 &{} \quad 0 \\ 1 &{} \quad 0 &{} \quad 0 \\ 0 &{} \quad 1 &{} \quad 0 \\ 0 &{} \quad 1 &{} \quad 0 \\ 0 &{} \quad 0 &{} \quad 1 \\ 0 &{} \quad 0 &{} \quad 1 \end{pmatrix} \begin{pmatrix} \beta _1 \\ \beta _2 \\ \beta _3 \end{pmatrix} + \begin{pmatrix} 1 &{} \quad 0 &{} \quad 0 &{} \quad 0 &{} \quad 0 &{} \quad 0 \\ 0 &{} \quad 0 &{} \quad 0 &{} \quad 1 &{} \quad 0 &{} \quad 0 \\ 0 &{} \quad 1 &{} \quad 0 &{} \quad 0 &{} \quad 0 &{} \quad 0 \\ 0 &{} \quad 0 &{} \quad 0 &{} \quad 0 &{} \quad 1 &{} \quad 0 \\ 0 &{} \quad 0 &{} \quad 1 &{} \quad 0 &{} \quad 0 &{} \quad 0 \\ 0 &{} \quad 0 &{} \quad 0 &{} \quad 0 &{} \quad 0 &{} \quad 1 \\ \end{pmatrix} \begin{pmatrix} p_{i1} \\ p_{i2} \\ p_{i3} \\ t_{i1} \\ t_{i2} \\ t_{i3} \end{pmatrix} \nonumber \\&\quad + \begin{pmatrix} 0 &{} \quad 0 &{} \quad 0 &{} \quad 1 &{} \quad 0 &{} \quad 0 \\ 1 &{} \quad 0 &{} \quad 0 &{} \quad 0 &{} \quad 0 &{} \quad 0 \\ 0 &{} \quad 0 &{} \quad 0 &{} \quad 0 &{} \quad 1 &{} \quad 0 \\ 0 &{} \quad 1 &{} \quad 0 &{} \quad 0 &{} \quad 0 &{} \quad 0 \\ 0 &{} \quad 0 &{} \quad 0 &{} \quad 0 &{} \quad 0 &{} \quad 1 \\ 0 &{} \quad 0 &{} \quad 1 &{} \quad 0 &{} \quad 0 &{} \quad 0 \\ \end{pmatrix} \begin{pmatrix} p_{j1} \\ p_{j2} \\ p_{j3} \\ t_{j1} \\ t_{j2} \\ t_{j3} \end{pmatrix} + \begin{pmatrix} 1 &{} \quad 0 &{} \quad 0 &{} \quad 0 &{} \quad 0 &{} \quad 0 \\ 0 &{} \quad 1 &{} \quad 0 &{} \quad 0 &{} \quad 0 &{} \quad 0 \\ 0 &{} \quad 0 &{} \quad 1 &{} \quad 0 &{} \quad 0 &{} \quad 0 \\ 0 &{} \quad 0 &{} \quad 0 &{} \quad 1 &{} \quad 0 &{} \quad 0 \\ 0 &{} \quad 0 &{} \quad 0 &{} \quad 0 &{} \quad 1 &{} \quad 0 \\ 0 &{} \quad 0 &{} \quad 0 &{} \quad 0 &{} \quad 0 &{} \quad 1 \\ \end{pmatrix} \begin{pmatrix} r_{ij1} \\ r_{ji1} \\ r_{ij2} \\ r_{ji2} \\ r_{ij3} \\ r_{ji3} \end{pmatrix} \end{aligned}$$

where $\varvec{X}$, $\varvec{Z}_{1}$, $\varvec{Z}_{2}$, and $\varvec{W}_{1}$ are the aforementioned design matrices that control in which row the mean, the person-level effects and the dyad-level effects appear.

We define the SEM part of the model for the vector of person effects and the vector of dyad effects, respectively. For the person effects, we assume that they are linear functions of $n_u$ latent person factors that are structurally related

8 $$\begin{aligned} \varvec{u}_i&= \varvec{\varLambda }_{\varvec{u}} \varvec{\eta }_{\varvec{u}_i} + \varvec{\varepsilon }_{\varvec{u}_i} \nonumber \\ \varvec{\eta }_{\varvec{u}_i}&= \varvec{B}_{\varvec{u}}\varvec{\eta }_{\varvec{u}_i} + \varvec{{\varGamma }}_{\varvec{u}}\varvec{x}_{\varvec{u}_i} + \varvec{\xi }_{\varvec{u}_i}, \end{aligned}$$

where $\varvec{\varLambda }_{\varvec{u}}$ is a $2k \times n_u$ matrix of factor loadings, $\varvec{\eta }_{\varvec{u}_i}$ is an $n_u \times 1$ vector of latent perceiver and target effects for person *i*, and $\varvec{\varepsilon }_{\varvec{u}_i}$ is a $2k \times 1$ vector of residual terms for this person. Furthermore, $\varvec{B}_{\varvec{u}}$ is an $n_u \times n_u$ matrix of structural coefficients describing the relations between the latent perceiver and target effects, and $\varvec{{\varGamma }}_{\varvec{u}}$ is a matrix of coefficients relating exogenous variables to the latent perceiver and target effects. Finally, $\varvec{\xi }_{\varvec{u}_i}$ is a vector of residual terms of the latent perceiver and target effects of person *i*; their distribution will be defined in the next section.

Any reader who is familiar with the standard SEM might just wonder why there are no latent factor means in the equation for the latent person effects $\varvec{\eta }_{\varvec{u}_i}$. The reasons for not including these means are that in order to estimate the parameters of this vector, an element of $\varvec{\beta }$ would have to be set to zero, and it would then have to be specified whether this observed variable mean should be modeled for the perceiver factor or for the target factor (otherwise, the mean structure of the model would not be identified). However, this decision is rather arbitrary, and therefore, we have decided not to consider latent factor means in our model. Of note, when a person-level variable (or dyad-level variable; see below) is considered in the model, the values in $\varvec{\beta }$ denote the values in the round-robin variable that are expected when the person-level variable is zero.

The same model is defined for the dyad effects. We again assume that the dyad effects are linear functions of $n_d$ structurally related latent dyad factors

9 $$\begin{aligned} \varvec{r}_d&= \varvec{\varLambda }_{\varvec{r}} \varvec{\eta }_{\varvec{r}_d} + \varvec{\varepsilon }_{\varvec{r}_d} \nonumber \\ \varvec{\eta }_{\varvec{r}_d}&= \varvec{B}_{\varvec{r}}\varvec{\eta }_{\varvec{r}_d} + \varvec{{\varGamma }}_{\varvec{r}}\varvec{x}_{\varvec{r}_d} + \varvec{\xi }_{\varvec{r}_d}. \end{aligned}$$

Here, $\varvec{\varLambda }_{\varvec{r}}$ is a $2k \times n_d$ matrix of factor loadings that relate the latent relationship vectors in the $n_d \times 1$ vector to the relationship effects of the *k* measures. $\varvec{\varepsilon }_{\varvec{r}_d}$ is a $2k \times 1$ vector of residual terms for dyad *d*. Finally, the structural part of the model consists of the $n_d \times n_d$ structural coefficient matrix $\varvec{B}_{\varvec{r}}$, the $\varvec{{\varGamma }}_{\varvec{r}}$ matrix of exogenous variable coefficients, and the vector of residual terms of the latent relationship effects $\varvec{\xi }_{\varvec{r}_d}$ for dyad *d*. We define the distribution of the residual terms in the next section.

To illustrate Eqs. 8 and 9, we take the same dyad again and assume that we have assessed three round-robin variables that reflect liking. If we presume that all three SRM effects indicate a respective *single* latent SRM effect, the equation for the person-level effects for person *i* is

10 $$\begin{aligned} \varvec{u}_i&= \begin{pmatrix} p_{i1} \\ p_{i2} \\ p_{i3} \\ t_{i1} \\ t_{i2} \\ t_{i3} \end{pmatrix} = \begin{pmatrix} 1 &{}\quad 0 \\ \lambda _{2p} &{} \quad 0 \\ \lambda _{3p} &{} \quad 0 \\ 0 &{} \quad 1 \\ 0 &{} \quad \lambda _{2t} \\ 0 &{} \quad \lambda _{3t} \end{pmatrix} \begin{pmatrix} \eta _{ui} \\ \eta _{vi} \end{pmatrix} + \begin{pmatrix} \varepsilon _{p_{i1}} \\ \varepsilon _{p_{i2}} \\ \varepsilon _{p_{i3}} \\ \varepsilon _{t_{i1}} \\ \varepsilon _{t_{i2}} \\ \varepsilon _{t_{i3}} \end{pmatrix} \nonumber \\ \varvec{\eta }_{\varvec{u}_i}&= \begin{pmatrix} \xi _{ui} \\ \xi _{vi} \end{pmatrix} \end{aligned}$$

whereby all other matrices are zero, and the first factor loading of each latent variable is set to one for identification purposes. Similarly, the equation for the dyad-level effects is

11 $$\begin{aligned} \varvec{r}_d&= \begin{pmatrix} r_{ij1} \\ r_{ji1} \\ r_{ij2} \\ r_{ji2} \\ r_{ij3} \\ r_{ji3} \end{pmatrix} = \begin{pmatrix} 1 &{} \quad 0 \\ 0 &{} \quad 1 \\ \lambda _{2,ij} &{} \quad 0 \\ 0 &{} \quad \lambda _{2,ji} \\ \lambda _{3,ij} &{} \quad 0 \\ 0 &{} \quad \lambda _{3,ji} \end{pmatrix} \begin{pmatrix} \eta _{ij,r_{d}} \\ \eta _{ji,r_{d}} \end{pmatrix} + \begin{pmatrix} \varepsilon _{r_{ij1}} \\ \varepsilon _{r_{ji1}} \\ \varepsilon _{r_{ij2}} \\ \varepsilon _{r_{ji2}} \\ \varepsilon _{r_{ij3}} \\ \varepsilon _{r_{ji3}} \\ \end{pmatrix} \nonumber \\ \varvec{\eta }_{\varvec{r}_d}&= \begin{pmatrix} \xi _{ij,r_{d}} \\ \xi _{ji,r_{d}} \end{pmatrix} \end{aligned}$$

Again, we set the first factor loading to one to identify the model, and all other matrices are defined to be zero. Please note that the measurement and structural parts for the relationship effects have to be defined for the relationship effects from *i* to *j* and the relationship effects from *j* to *i*. Although our model framework allows different models to be defined for the two types of relationship effects, we do not believe this is meaningful because it would, for example, suggest that the factor structure for the liking judgments of individual *i* about individual *j* differs from the factor structure for the liking judgments of *j* about *i*. Furthermore, it would contradict the assumption of the SRM that the dyads are indistinguishable (Kenny et al. 2006; Nestler 2018). In the following, we therefore assume that a researcher has defined the same measurement model and the same structural model for the two types of relationship effects. Furthermore, we also assume that the error terms of the person-level effects and the relationship effects are correlated in a certain way (see below), and this is consistent with the model used for the illustration here.

### Covariance Structure and Mean Structure

Equations 6, 8, and 9 can be used to derive the covariance structure and the mean structure of the whole round-robin vector $\varvec{y}$ conditional on the covariates contained in $\varvec{x}_{\varvec{u}_i}$ or in $\varvec{x}_{\varvec{r}_d}$. We assume that $\varvec{\varepsilon }_{\varvec{u}_i}$, $\varvec{\xi }_{\varvec{u}_i}$, $\varvec{\varepsilon }_{\varvec{r}_d}$, and $\varvec{\xi }_{\varvec{r}_d}$ are (multivariate normal distributed; see below) random variables that have an expectation of zero and covariance matrix $\varvec{\varPhi }_{ \varvec{u}}$, $\varvec{\varPsi }_{ \varvec{u}}$, $\varvec{\varPhi }_{ \varvec{r}}$, and $\varvec{\varPsi }_{ \varvec{r}}$. In the following, we adhere to common practice and omit the conditioning on the covariates when we state the model assumptions.

Using standard results from the SEM literature (e.g., Bollen 1989; Lee 2007; Mulaik 2009), we first find that the covariance matrix of the person-level effects is

12 $$\begin{aligned} \varvec{{{\varSigma }}}_{ \varvec{u}} = \varvec{\varLambda }_{ \varvec{u} } (\varvec{I} - \varvec{B}_{\varvec{u}})^{-1} \varvec{\varPhi }_{ \varvec{u}} (\varvec{I} - \varvec{B}_{\varvec{u}})'^{-1} \varvec{\varLambda }_{ \varvec{u} } ' + \varvec{\varPsi }_{ \varvec{u}} \end{aligned}$$

and that the covariance matrix of the dyad-level effects is

13 $$\begin{aligned} \varvec{{{\varSigma }}}_{\varvec{r}} = \varvec{\varLambda }_{ \varvec{r} } (\varvec{I} - \varvec{B}_{\varvec{r}})^{-1} \varvec{\varPhi }_{ \varvec{r}} (\varvec{I} - \varvec{B}_{\varvec{r}})'^{-1} \varvec{\varLambda }_{ \varvec{r} } ' + \varvec{\varPsi }_{ \varvec{r}} . \end{aligned}$$

Here, $\varvec{I}$ is an identity matrix of respective size, $\varvec{\varPhi }_{u}$ and $\varvec{\varPhi }_{r}$ are the covariance matrices of the latent person effects and the latent dyad effects, respectively, and $\varvec{\varPsi }_{u}$ and $\varvec{\varPsi }_{r}$ denote the covariance matrices of the respective residual terms. Similarly, standard results from the SEM literature imply that the expectation of the person-level effects is

14 $$\begin{aligned} \varvec{\mu }_{ \varvec{u_i} } = \varvec{\varLambda }_{\varvec{u}} (\varvec{I} - \varvec{B}_{\varvec{u}})^{-1} \varvec{{\varGamma }}_{\varvec{u}}\varvec{x}_{\varvec{u}_i} , \end{aligned}$$

and that the expectation of the dyad-level effects is

15 $$\begin{aligned} \varvec{\mu }_{ \varvec{r_d} } = \varvec{\varLambda }_{\varvec{r}} (\varvec{I} - \varvec{B}_{\varvec{r}})^{-1} \varvec{{\varGamma }}_{\varvec{r}}\varvec{x}_{\varvec{r}_d} . \end{aligned}$$

Again, we will use our running example to illustrate Eqs. 12 and 14 (the illustration for the dyad-level effect equations would be very similar). In this case (i.e., three round-robin variables, one latent perceiver-effect factor, and one latent target-effect factor) $\varvec{{{\varSigma }}}_{\varvec{u}}$ is

16 $$\begin{aligned} \begin{pmatrix} 1 &{} \quad 0 \\ \lambda _{2p} &{} \quad 0 \\ \lambda _{3p} &{} \quad 0 \\ 0 &{} \quad 1 \\ 0 &{} \quad \lambda _{2t} \\ 0 &{} \quad \lambda _{3t} \end{pmatrix} \begin{pmatrix} \phi ^2_{t} &{} \quad \phi _{pt} \\ \phi _{uv} &{} \quad \phi ^{2}_{t} \end{pmatrix} \begin{pmatrix} 1 &{} \quad 0 \\ \lambda _{2p} &{} \quad 0 \\ \lambda _{3p} &{} \quad 0 \\ 0 &{} \quad 1 \\ 0 &{} \quad \lambda _{2t} \\ 0 &{} \quad \lambda _{3t} \end{pmatrix}^{T} + \begin{pmatrix} \psi ^2_{p1} &{} \quad 0 &{} \quad 0 &{} \quad \psi _{pt1} &{} \quad 0 &{} \quad 0 \\ 0 &{} \quad \psi ^2_{p2} &{} \quad 0 &{} \quad 0 &{} \quad \psi _{pt2} &{} \quad 0 \\ 0 &{} \quad 0 &{} \quad \psi ^2_{p3} &{} \quad 0 &{} \quad 0 &{} \quad \psi _{pt3} \\ \psi _{pt1} &{} \quad 0 &{} \quad 0 &{} \quad \psi ^2_{t1} &{} \quad 0 &{} \quad 0 \\ 0 &{} \quad \psi _{pt2} &{} \quad 0 &{} \quad 0 &{} \quad \psi ^2_{t2} &{} \quad 0 \\ 0 &{} \quad 0 &{} \quad \psi _{pt3} &{} \quad 0 &{} \quad 0 &{} \quad \psi ^2_{t3} \end{pmatrix} \end{aligned}$$

and $\varvec{\mu }_{\varvec{u_i}}$ is zero. Note that $\psi _{ptk}$ is the aforementioned term representing the covariance between the person-level error terms. It describes the covariance between the perceiver and target effects for measure *k* not explained by the covariance of the perceiver-effect factor and the target-effect factor.

Assuming that the person-level effects and the dyad-level effects in Eq. 6 are independent, it follows that the covariance matrix of the round-robin vector $\varvec{y}$ can be calculated as

17 $$\begin{aligned} \varvec{{{\varSigma }}}_{ \varvec{y} } =&\sum _{i=1}^I \varvec{Z}_i \varvec{{{\varSigma }}}_{ \varvec{u} } \varvec{Z}_i ' + \sum _{d=1}^D \varvec{W}_d \varvec{{{\varSigma }}}_{\varvec{r}} \varvec{W}_d ' \nonumber \\ =&\sum _{i=1}^I \varvec{Z}_i (\varvec{\varLambda }_{ \varvec{u} } (\varvec{I} - \varvec{B}_{\varvec{u}})^{-1} \varvec{\varPhi }_{ \varvec{u}} (\varvec{I} - \varvec{B}_{\varvec{u}})'^{-1} \varvec{\varLambda }_{ \varvec{u} } ' + \varvec{\varPsi }_{ \varvec{u}}) \varvec{Z}_i ' \nonumber \\&+ \sum _{d=1}^D \varvec{W}_d (\varvec{\varLambda }_{ \varvec{r} } (\varvec{I} - \varvec{B}_{\varvec{r}})^{-1} \varvec{\varPhi }_{ \varvec{r}} (\varvec{I} - \varvec{B}_{\varvec{r}})'^{-1} \varvec{\varLambda }_{ \varvec{r} } ' + \varvec{\varPsi }_{ \varvec{r}}) \varvec{W}_d ' \end{aligned}$$

Furthermore, the expectation of $\varvec{y}$ is

18 $$\begin{aligned} \varvec{\mu }_{ \varvec{y} } =&\varvec{X} \varvec{\beta } + \sum _{i=1}^I \varvec{Z}_i \varvec{\varLambda }_{\varvec{u}} (\varvec{I} - \varvec{B}_{\varvec{u}})^{-1} \varvec{{\varGamma }}_{\varvec{u}}\varvec{x}_{\varvec{u}_i} \nonumber \\&+ \sum _{d=1}^D \varvec{W}_d \varvec{\varLambda }_{\varvec{d}} (\varvec{I} - \varvec{B}_{\varvec{d}})^{-1} \varvec{{\varGamma }}_{\varvec{d}}\varvec{x}_{\varvec{r}_d} . \end{aligned}$$

In summary, our framework for combining the SRM and the SEM is based on writing the round-robin judgments in terms of the person-level effects of all individuals *I* and the dyad-level effects of all dyads *D*. This allows us to define a measurement model and a structural model for the two types of SRM effects. The covariance structure and the mean structure of the whole round-robin judgment vector can then be obtained by using standard results mentioned in the SEM literature. Note that the univariate SRM described in the Introduction is a special case of the suggested model in which the described results are obtained when the following parameters are set to zero: the two factor loading matrices, $\varvec{\varLambda }_{\varvec{u}}$ and $\varvec{\varLambda }_{\varvec{d}}$, the covariance matrices of the latent SRM effects, $\varvec{\varPhi }_{\varvec{u}}$ and $\varvec{\varPhi }_{\varvec{d}}$, and the matrices containing the latent regression coefficients, $\varvec{B}_{\varvec{u}}$, $\varvec{B}_{\varvec{d}}$, $\varvec{{\varGamma }}_{\varvec{u}}$, and $\varvec{{\varGamma }}_{\varvec{d}}$. In a similar vein, it is possible to show that the multivariate SRM is a special case of the SR-SEM suggested here.

### Estimation of the SR-SEM Parameters

Let $\varvec{\theta }$ be the vector of the SR-SEM parameters that we want to estimate with the data. Here, we suggest utilizing the maximum likelihood (ML) method to obtain $\varvec{\hat{\theta }}$ with empirical data. To this end, we assume that all latent variables are multivariate normally distributed (see also Bollen 1989) and that their expectation is zero. From this, it follows that the vector of round-robin judgments is also multivariate normal with expectation $\varvec{\mu }_{ \varvec{y} }$ and covariance matrix $\varvec{{{\varSigma }}}_{ \varvec{y} }$ as given in Eqs. 18 and 14, respectively.

This result can be used to obtain the log-likelihood of the data given the parameter vector $\varvec{\theta }$ (ignoring constant terms)

19 $$\begin{aligned} l(\varvec{\theta }) \propto - \frac{1}{2} \log (|\varvec{{{\varSigma }}}_{ \varvec{y} }^{-1}|) - \frac{1}{2} (\varvec{y} - \varvec{\mu }_{\varvec{y}})^{T} \varvec{{{\varSigma }}}_{ \varvec{y} }^{-1} (\varvec{y} - \varvec{\mu }_{\varvec{y}}) \end{aligned}$$

where the covariance matrix and the vector of expectations are functions of the unknown parameter vector $\varvec{\theta }$.

Equation 19 can be used to derive the score equation and the expected Fisher information matrix (see e.g., Magnus and Neudecker 1999). The score equation is

20 $$\begin{aligned} \frac{\partial l}{ \partial \theta _h} = - \frac{1}{2} \mathrm {tr} \left( \varvec{{{\varSigma }}} _{\varvec{y} }^{-1} \frac{\partial \varvec{{{\varSigma }}} _{\varvec{y} } }{\partial \theta _h} \right) + \frac{1}{2} (\varvec{y} - \varvec{\mu }_{\varvec{y}})^{T} \varvec{{{\varSigma }}}_{\varvec{y} }^{-1} \frac{\partial \varvec{{{\varSigma }}}_{\varvec{y} } }{\partial \theta _h} \varvec{{{\varSigma }}}_{\varvec{y} }^{-1} (\varvec{y} - \varvec{\mu }_{\varvec{y}}) + (\varvec{y} - \varvec{\mu }_{\varvec{y}})^{T} \varvec{{{\varSigma }}}_{\varvec{y} }^{-1} \frac{\partial \varvec{\mu }_{\varvec{y} } }{\partial \theta _h} \end{aligned}$$

and the expected information matrix is

21 $$\begin{aligned} \frac{\partial l^2}{ \partial \theta _h \theta _k} = - \frac{1}{2} \mathrm {tr} \left( \varvec{{{\varSigma }}} _{\varvec{y} }^{-1} \frac{\partial \varvec{{{\varSigma }}} _{\varvec{y} } }{\partial \theta _h} \varvec{{{\varSigma }}} _{\varvec{y} }^{-1} \frac{\partial \varvec{{{\varSigma }}} _{\varvec{y} } }{\partial \theta _k} \right) + \left( \frac{\partial \varvec{\mu } _{\varvec{y} } }{\partial \theta _h} \right) ' \varvec{{{\varSigma }}} _{\varvec{y} }^{-1} \left( \frac{\partial \varvec{\mu } _{\varvec{y} } }{\partial \theta _k} \right) . \end{aligned}$$

We use the score equation and the expected information matrix to implement a Fisher Scoring algorithm (see Pawitan 2001, for an introduction). For the algorithm, we have to compute the derivatives of $\varvec{{{\varSigma }}}_{\varvec{y}}$ and the derivatives of $\varvec{\mu } _{\varvec{y} }$. However, this can be reduced to finding the derivatives of $\varvec{{{\varSigma }}}_{\varvec{u}}$, $\varvec{{{\varSigma }}}_{\varvec{r}}$, $\varvec{\mu }_{\varvec{u}}$, and $\varvec{\mu }_{\varvec{r}}$ as

22 $$\begin{aligned} \frac{\partial \varvec{{{\varSigma }}}_{\varvec{y} } }{\partial \theta _h} = \sum _{i=1}^I \varvec{Z}_i \frac{\partial \varvec{{{\varSigma }}}_{ \varvec{u} } }{\partial \theta _h} \varvec{Z}_i^{T} + \sum _{d=1}^D \varvec{W}_d \frac{\partial \varvec{{{\varSigma }}}_{\varvec{r} } }{\partial \theta _h} \varvec{W}_{d}^{T} \end{aligned}$$

and

23 $$\begin{aligned} \frac{\partial \varvec{\mu }_{\varvec{y} } }{\partial \theta _h} = \varvec{X} \frac{\partial \varvec{\beta } }{\partial \theta _h} + \sum _{i=1}^I \varvec{Z}_i \frac{\partial \varvec{\mu }_{ \varvec{u} } }{\partial \theta _h} + \sum _{d=1}^D \varvec{W}_d \frac{\partial \varvec{\mu }_{\varvec{r} } }{\partial \theta _h} \end{aligned}$$

follows from the linearity of differentiation. Thus, computing the derivatives of $\varvec{{{\varSigma }}}_{\varvec{y}}$ and $\varvec{\mu } _{\varvec{y} }$, respectively, can be reduced to finding the partial derivatives of $\varvec{{{\varSigma }}}$ and $\varvec{\mu }$ for the person-level or dyad-level effects with respect to the elements in $\varvec{\varLambda }$, $\varvec{B}$, $\varvec{\varPhi }$, $\varvec{\varTheta }$, and $\varvec{{\varGamma }}$. However, these derivatives can be found in the SEM literature (e.g., Mulaik 2009, see also Appendix B where we provide these formulas).

In summary, we utilized an ML approach to estimate the parameters of the SR-SEM. The parameters of the model were obtained with a Fisher Scoring algorithm that used the first derivatives and the expected information matrix of the log-likelihood function. All functions and algorithms that we used are implemented in the R package srm, which can be downloaded from https://github.com/alexanderrobitzsch/srm.

## Illustrative Examples

We will use part of the data from the EXACT study (see https://osf.io/67m4y/, Study 2, Niemeyer et al. 2018) to illustrate the SR-SEM. The EXACT study was conducted to investigate the processes underlying accurate personality judgments. To this end, participants took part in a zero-acquaintance round-robin group experiment consisting of two group sessions. At each session, the group members of twenty-four round-robin groups (consisting of five to six individuals each, overall *n* = 141) were asked to introduce themselves briefly to their round-robin group members. Thereafter, they were asked to judge the other group members on 10 adjectives encompassing different interpersonal personality dimensions (Jacobs and Scholl 2005). All judgments were assessed on 9-point Likert-type scales (ranging from 1 = not at all to 9 = very much). Here, we used four round-robin judgments-shy, nervous, calm, and insightful-to compute a one-factor SR-CFA and a SR-path model.

For the SR-CFA, a one-factor model was defined for the three round-robin judgments shy, nervous, and calm. We assumed that the perceiver effects contained in the three judgments would load on one latent perceiver-effect factor reflecting neuroticism. The same one-factor model was also posited for the targets effects. In addition, we assumed that the two latent factors were correlated, and we allowed the perceiver-effect and target-effect residual terms of a certain round-robin variable to be correlated. Finally, a one factor model was also defined for the relationship effects. For the SR-path model, we used the perceiver and target effects of the round-robin variable calm to predict the perceiver and target effects of the round-robin judgment insightful. We did not specify a path model for the relationship effects, but estimated a model in which the variance and the covariance parameter between the relationship effects for each of the two variables were estimated.

We used our R package srm to obtain the ML parameter estimates. We also used a two-step approach to estimate the factor models. To this end, we estimated the perceiver effects, target effects, and relationship effects of the three round-robin variables using the R package TripleR (Schönbrodt et al. 2017). The person-level effects were then used as items in a respective CFA model or path model. Both models were fit in R using the package lavaan (Rosseel 2012). lavaan was also used to estimate the CFA model and the saturated model for the relationship effects. All *R* codes as well as the data can be downloaded from the Open Science Framework (OSF, https://osf.io/9twkm/).

**Table 1:** Selected results for the SR-CFA

	2-Step		SR-SEM	
Parameter	Estimate	SE	Estimate	SE
$\lambda _{21p}$	1.56	0.18	1.89	0.49
$\lambda _{31p}$	1.17	0.14	1.21	0.29
$\lambda _{21t}$	0.95	0.07	0.85	0.09
$\lambda _{31t}$	0.77	0.06	0.71	0.08
$\phi ^{2}_{p}$	0.35	0.08	0.18	0.09
$\phi ^{2}_{t}$	1.33	0.23	1.23	0.27
$\phi _{pt}$	$-$0.03 ($-$0.04)	0.06	$-$0.14 ($-$0.29)	0.08
$\lambda _{21r}$	1.35	0.10	1.35	0.12
$\lambda _{31r}$	1.13	0.08	1.13	0.09
$\phi ^{2}_{r}$	0.58	0.07	0.95	0.14
$\phi _{rr}$	$-$0.05 ($-$0.09)	0.04	$-$0.01 ($-$0.01)	0.08

*Note.* Unstandardized parameter estimates are shown in the table. The value in parentheses appearing after a covariance parameter represents the respective correlation. The residual variance terms of the person-level effect error terms and dyad-level effect error terms, respectively, as well as the covariance parameters between the person-level or dyad-level residual error terms of an item are not shown in the table. SE = standard error

Results for the SR-CFA Table 1 shows the parameter estimates that we obtained with the two-step approach and the SR-CFA for the two-factor model of the person-level effects. For both approaches, the (unstandardized) factor loadings were moderate to high with higher loadings for the perceiver effects compared with the target effects. The SR-CFA approach yielded smaller variance estimates for the latent factors than the two-step approach. For both estimators, the terms were greater than zero. This indicates that people differed in their average perception of others concerning neuroticism and also in how they were judged on this dimension on average. Finally, we found a small negative correlation between the neuroticism perceiver effect and the neuroticism target effect factors with the SR-CFA approach, but not with the two-step approach. This correlation indicates that people who judge others as neurotic on average are judged to be less neurotic on average. Finally, for all parameters, we found larger standard errors for the SR-CFA approach compared to the two-step approach.

Table  1 also displays the results for the dyad-level effects. Again, the two estimation approaches yielded very similar results for the factor loadings. The relationship variance was greater for the SR-CFA compared to the two-step approach. Overall, a large part of the round-robin rating variance of a specific item can be attributed to dyad-specific perceptions. Interestingly, the latent relationship effects were only weakly correlated, indicating that there is no tendency to reciprocate unique nervousness perceptions. Finally, the standard errors of the parameters were greater in case of the SR-CFA approach compared to the two-step approach, but the differences were smaller than the differences in the standard errors for the person-level effects.

**Table 2:** Selected results for the SR-path model

	2-Step		SR-SEM	
Parameter	Estimate	SE	Estimate	SE
$b_{p_{in},p_{ca}}$	$-$0.34	0.07	$-$0.77	0.25
$b_{t_{in},t_{ca}}$	$-$0.05	0.07	$-$0.22	0.22
$b_{p_{in},t_{ca}}$	0.05	0.06	0.14	0.18
$b_{t_{in},p_{ca}}$	$-$0.21	0.07	$-$0.21	0.25
$\phi ^{2,ca}_{r}$	1.43	0.08	1.71	0.12
$\phi ^{2,in}_{r}$	1.06	0.06	2.28	0.15
$\phi ^{ca}_{rr}$	$-$0.06 ($-$0.06)	0.08	0.16 (0.10)	0.12
$\phi ^{in}_{rr}$	0.04 ( 0.04)	0.06	0.04 (0.02)	0.15

*Note.* Unstandardized parameter estimates are shown in the table. SE = standard error, ca = calm, in = insightful

Results for the SR-path model In this model, we regressed the perceiver and target effects of the insightful judgments on the perceiver and target effects of the calm judgments (reverse-coded). Table 2 shows the path coefficients that we obtained with the two-step approach and the SR-path model. The results show that the SR-SEM coefficients were higher in three out of four cases (the exception is $b_{t_{in},p_{ca}}$). For both models, the coefficient describing the relation between the two perceiver effects was significantly different from zero (i.e., $b_{p_{in},p_{ca}}$). This indicates that participants who judged others to be more unstressed on average, also judged others to be less insightful on average. Again, for all parameters, we found larger standard errors for the SR-path model compared to the two-step approach. This has the consequence that $b_{t_{in},p_{ca}}$, describing the relation between the target effects of insightful and the perceiver effects of calm, is significantly different from zero for the two-step approach but not for the SR-path model.

For the relationship effects, we found that the relationship variance was greater for the SR-path model compared to the two-step approach. The standard errors of the SR-path model were also greater. Again, the results indicate that a large part of the round-robin rating variance of a specific item can be attributed to dyad-specific perceptions.

In summary, the results of the two examples show that the two approaches can produce partly similar but also different parameter estimates. Furthermore, the standard errors of the two-step approach are smaller than the standard errors of the SR-SEM. A potential explanation for the differences in the parameter estimates may be that we used a data set with only a few round-robin groups. Thus, the resulting coefficients could be biased by both methods (see e.g., Lüdtke et al. 2013, 2018; Nestler 2018). The differences in the standard errors might be the result of the higher variability of the ML estimator in case of a few round-robin groups. In addition, the standard errors of the two-step approach might be underestimated because they did not take into account the uncertainty from computing the first step estimates. In the next section, we report the results of a small simulation study that was done to examine the suitability of these explanations.

## Simulation Study

Specifically, we conducted a small simulation study to compare the performance of the ML approach with the two-step approach for estimating the parameters of an SR-CFA (see the first illustration) with different numbers of round-robin groups and different numbers of round-robin group members.

Population model and simulation conditions We simulated an SR-CFA model in which we assumed that one latent perceiver-effect factor, one latent target-effect factor, and one latent relationship-effect factor determined the responses to three round-robin variables. For the person-level effects, the model matrices were set to the following values:

$$\begin{aligned} \varvec{\varLambda }_{\varvec{u}}^T = \begin{pmatrix} 1.0 &{} \quad 1.2 &{} \quad 0.7 &{} \quad 0.0 &{} \quad 0.0 &{} \quad 0.0 \\ 0.0 &{} \quad 0.0 &{} \quad 0.0 &{} \quad 1.0 &{} \quad 0.6 &{} \quad 0.6 \\ \end{pmatrix} , \varvec{\varPhi }_{\varvec{u}} = \begin{pmatrix} 0.40 &{} \quad 0.05 \\ 0.05 &{} \quad 0.20 \\ \end{pmatrix} \end{aligned}$$

and

$$\begin{aligned} \varvec{\varPsi }_{\varvec{u}} = \begin{pmatrix} 0.20 &{} \quad 0.00 &{} \quad 0.00 &{} \quad 0.05 &{} \quad 0.00 &{} \quad 0.00 \\ 0.00 &{} \quad 0.20 &{} \quad 0.00 &{} \quad 0.00 &{} \quad 0.00 &{} \quad 0.00 \\ 0.00 &{} \quad 0.00 &{} \quad 0.20 &{} \quad 0.00 &{} \quad 0.00 &{} \quad 0.03 \\ 0.05 &{} \quad 0.00 &{} \quad 0.00 &{} \quad 0.10 &{} \quad 0.00 &{} \quad 0.00 \\ 0.00 &{} \quad 0.00 &{} \quad 0.00 &{} \quad 0.00 &{} \quad 0.10 &{} \quad 0.00 \\ 0.00 &{} \quad 0.00 &{} \quad 0.03 &{} \quad 0.00 &{} \quad 0.00 &{} \quad 0.10 \\ \end{pmatrix} \end{aligned}$$

For the dyad-level effects, the matrices were set to:

24 $$\begin{aligned} \varvec{\varLambda }_{\varvec{r}}^T = \begin{pmatrix} 1.0 &{} \quad 0.0 &{} \quad 0.8 &{} \quad 0.0 &{} \quad 1.4 &{} \quad 0.0 \\ 0.0 &{} \quad 1.0 &{} \quad 0.0 &{} \quad 0.8 &{} \quad 0.0 &{} \quad 1.4 \\ \end{pmatrix} , \varvec{\varPhi }_{\varvec{r}} = \begin{pmatrix} 0.60 &{} \quad 0.15 \\ 0.15 &{} \quad 0.60 \\ \end{pmatrix} \end{aligned}$$

and

$$\begin{aligned} \varvec{\varPsi }_{\varvec{r}} = \begin{pmatrix} 0.30 &{} \quad 0.00 &{} \quad 0.00 &{} \quad 0.00 &{} \quad 0.00 &{} \quad 0.00 \\ 0.00 &{} \quad 0.30 &{} \quad 0.00 &{} \quad 0.00 &{} \quad 0.00 &{} \quad 0.00 \\ 0.00 &{} \quad 0.00 &{} \quad 0.50 &{} \quad 0.10 &{} \quad 0.00 &{} \quad 0.00 \\ 0.00 &{} \quad 0.00 &{} \quad 0.10 &{} \quad 0.50 &{} \quad 0.00 &{} \quad 0.00 \\ 0.00 &{} \quad 0.00 &{} \quad 0.00 &{} \quad 0.00 &{} \quad 0.40 &{} \quad -0.20 \\ 0.00 &{} \quad 0.00 &{} \quad 0.00 &{} \quad 0.00 &{} \quad -0.20 &{} \quad 0.40 \\ \end{pmatrix}. \end{aligned}$$

The specification of the population parameters for the variance and covariance terms followed previous SRM research (see Kenny 1994; Kenny et al. 2006, for summaries) showing, for instance, that the variance of the individual level effects is lower than the variance of the dyad level effects, and that the perceiver variance is higher than the target variance. Furthermore, the perceiver and target effects and the relationship effects are typically positively correlated. For these reasons, we set the variance of the latent relationship factors to 0.60 and their covariance to 0.15 (implying a correlation of about 0.10), and the variance of the perceiver effect factor to 0.40, of the target effect factor to 0.20, and their covariance to 0.05 (implying a correlation of about 0.20). The values for the variance and the covariance parameters of the residual terms were based on similar considerations, although we set some of the specific covariance terms to zero or negative values to examine the generalizability of the simulation results. Finally, the pattern of factor loadings were inspired by the result pattern of the illustrative example.

To examine the properties of the two estimators, we also manipulated the number of round-robin groups and the number of round-robin group members: The number of round-robin groups was 15, 50, or 100, and the number of round-robin group members was either 5, 10, or 15. We note that $5 \cdot 15 = 75$ can be considered a very small sample size for latent variable models that use ML estimation (Mulaik 2009). Although we do not expect that the asymptotic properties for ML estimation will be satisfied in this condition, we included it as we believe that it is an interesting comparison condition. For each of the nine simulation conditions, 1000 samples were drawn from the population.

**Table 3:** Relative bias in percent (RB), relative root mean square error (RMSE), and coverage rate (CR) for the ML estimator and the two-step approach as a function of the number of round-robin groups *G* and the number of persons within each round-robin group *n*

		RB		RMSE		CR	
*n*	*G*	ML	2-Step	ML	2-Step	ML	2-Step
*Factor variance perceiver effects (true value = 0.4)*							
5	15	−1	20	0.46	0.40	88.8	90.0
	50	0	21	0.24	0.28	93.5	74.9
	100	−1	21	0.16	0.24	95.0	58.0
10	15	1	10	0.23	0.23	93.9	92.0
	50	0	10	0.12	0.15	94.5	86.9
	100	0	10	0.09	0.13	94.4	75.9
15	15	−1	6	0.17	0.17	94.6	94.4
	50	0	6	0.09	0.11	94.8	89.2
	100	0	6	0.07	0.09	94.8	82.4
*Factor variance target effects (true value = 0.2)*							
5	15	2	44	1.07	0.63	75.4	82.0
	50	6	41	0.51	0.47	90.5	52.7
	100	2	41	0.32	0.44	94.2	22.3
10	15	1	14	0.38	0.29	92.7	91.3
	50	1	15	0.20	0.21	95.0	79.1
	100	1	16	0.14	0.18	95.1	62.1
15	15	0	8	0.25	0.21	94.9	93.7
	50	0	9	0.13	0.13	96.0	88.1
	100	0	9	0.10	0.12	94.9	77.0
*Loading third item perceiver effect (true value = 0.7)*							
5	15	−1	22	0.29	0.32	90.2	79.8
	50	0	22	0.15	0.25	94.3	47.4
	100	0	22	0.10	0.24	94.9	18.9
10	15	0	11	0.14	0.17	93.9	87.7
	50	0	10	0.08	0.13	95.3	68.4
	100	0	11	0.06	0.12	94.7	44.0
15	15	0	7	0.11	0.13	95.2	89.0
	50	0	7	0.06	0.09	95.3	78.1
	100	0	7	0.04	0.08	95.5	56.8

Estimators The ML estimates were obtained using the srm package. To obtain the parameters with the two-step approach, we first estimated the perceiver effects, target effects, and relationship effects of round-robin variables using the R package TripleR (Schönbrodt et al. 2017). Thereafter, we fitted the respective CFA models using the lavaan package (Rosseel 2012).

Dependent measures We used the relative percentage bias (RB) of the parameter estimates, the relative root mean square error (RMSE), and the coverage rate to investigate the performance of the two approaches. To decrease the influence of extreme parameter estimates, we employed robust measures of the average and the standard deviation of the estimates of a parameter in a simulation condition to compute the RB and the relative RMSE (i.e., the median and the squared median absolute deviation; see Talloen et al. 2019). The RB was then computed by taking the difference between the median of a parameter in a simulation condition and the true parameter. Thereafter, this difference was divided by the true parameter (i.e., $(\mathrm{Med}({\hat{\theta }}) - \theta )/\theta$, where $\theta$ is the true parameter value). To compute the relative RMSE, we first computed the sum of the squared bias and the squared median absolute deviation for the parameter in the respective simulation condition. Thereafter, we divided this sum by the absolute value of the true population parameter. The observed coverage of the 95% confidence intervals was determined by computing the confidence interval with the standard error of an estimate in each replication. The coverage was then coded 1 if the true parameter value was included in the interval and 0 if the true parameter was not.

Results Table 3 presents the results of the simulation study for the factor variance of the actor effects, the factor variance of the partner effects, and the loading of the third item of the partner effect factor. The results for the other parameters are very similar to the results of the three selected parameters and were excluded here for space reasons. In the accompanying OSF project (see https://osf.io/9twkm/), we provide the results on the bias, RMSE, and coverage of the other SRM parameter estimates. As can be seen in Table 3, the ML approach yielded acceptable biases and coverage rates in all simulation conditions except in the case of few small round robin groups. The two-step approach yielded substantial parameter estimate bias in almost all simulation conditions, particularly when the group size was small and the estimated person-level effects are not very reliable estimates of the true individual effects. Also, the approach leads to undercoverage in most conditions. The RMSE results showed that independent upon the estimation approach, it is more advantageous to use fewer large round-robin groups instead of many smaller round-robin groups. Furthermore, the two-step approach seems to be more stable (i.e., has a smaller relative RMSE) in some conditions, although it is more biased. However, with a large number of groups (i.e., *G* = 100) the ML approach is more efficient and clearly outperforms the two-step approach even in conditions with small group sizes. This shows that a substantial number of groups is needed for ML estimation to show its superior asymptotic properties.

## Discussion

With the present paper, our goal was to present a combination of the social relations model (SRM) with structural equation models (SEMs). The basis for this combination was to write the round-robin judgments in terms of the person effects for the different individuals and the relationship effects of the different dyads. This presentation allowed us to derive the mean and the covariance structure of the round-robin judgments. These moments were then used to derive an ML estimator, which we implemented in the open-source and freely available R package srm. Finally, the results of the simulation study showed that the suggested ML estimator has good statistical properties.

Our SR-SEM has a number of advantages in comparison with earlier approaches that have been suggested for the SRM: First, our model can be used to examine structural hypotheses concerning multivariate SRM effects. This offers applied researchers a number of new modeling options. For instance, researchers can use the SR-SEM to conduct SRM path analyses in which SRM effects are regressed on other SRM effects or exogenous covariates. Furthermore, researchers can now compute SR-CFAs to test hypotheses concerning the factor structure of certain SRM effects (e.g., Srivastava et al. 2010; Wood et al. 2010) or to model longitudinal SRM data (Nestler et al. 2017). Previously, researchers have typically employed a two-step approach that is statistically problematic (but see Lüdtke et al. 2018), but our framework will allow them to use a one-step approach that is more efficient. Second, by using a maximum likelihood estimator, standard errors can be derived so that established significance tests for all model components are available. Finally, although we did not explicitly mention it here, our ML approach is a full-information maximum likelihood approach that allows for missing data in the round-robin variables and the exogenous covariates. It is an important topic for future research to evaluate the potential of the ML approach for dealing with incomplete round-robin ratings and covariates (see also Jorgensen et al. 2018).

The model presented here extends earlier models for multivariate SRM data. Nestler (2018), for example, presented a maximum likelihood approach and a restricted maximum likelihood approach for multiple round-robin variables; these were based on integrating the SRM into a mixed-model framework (see McCulloch et al. 2004). However, such approaches are limited because they cannot be used to estimate more complicated models such as SR-path models or SR-CFAs. Another approach was suggested by Mehta (2013a) and is also based on a mixed-model framework. It can be used to estimate multivariate SRMs (Mehta 2018) and is implemented in the R package xxM (Mehta 2013b). We believe that comparing the two approaches will be an interesting task for future research.

Although we believe that the SR-SEM proposed here is already a very complex and flexible model, we believe that it can be extended in future research to make it as general as the SEM of Muthén (2002). First, in its present form the SR-SEM does not allow to model exogenous effects on person-level components in $\varvec{u}_i$ (see Eq. 8) or dyad-level components in $\varvec{r}_i$ (see Eq. 9). In some research contexts, this might be an interesting extension, such as when one is interested to investigate whether exogenous variables moderate person-level SRM effects (Kenny 1994). Alternatively, when the SR-SEM were to be used for longitudinal SRM data in the future (see below), researchers might be interested in examining the effect of time-varying person variables. Furthermore, the model does not contain a representation for multiple-indicator exogenous variables (e.g., latent personality traits), although there are research contexts in which this component might be interesting. In the srm package, it is indirectly possible to model such factors, but in future research, this should be implemented in a more user-friendly way.^2^

Another task for future research is to consider non-normal data. Here, we assumed that all latent variables were normally distributed. When this assumption is not met, incorrect standard errors may result. A simple approach would be to correct the standard errors using the Huber–White sandwich estimator; however, future research has to determine the performance of this correction for multivariate SRM data. Another interesting task for future research is to extend our approach to multiple groups. This would allow researchers to explore whether all or some of the parameters are group specific; for instance, whether the results of our SR-CFA model differ between women and men (Card et al. 2005; Lüdtke et al. 2013). A problem with this extension is that in the context of the SRM, “groups” can refer to the nature of the assessed round-robin groups or to groups of individuals appearing *within* the round-robin groups. That is, one can assess round-robin groups consisting only of women or men, or one may have round-robin groups available in which the women and men are mixed. The first type of multiple groups is unproblematic for the ML approach suggested here because the independence of the round-robin groups means that the likelihood contributions of the different groups can be summed (as is done in multiple-group SEMs; Bollen 1989). However, the second type of “groups” is more challenging because the data between the “groups” is nested within round-robin groups and is therefore dependent (see also Ryu 2015).

Another interesting avenue for future research is to apply the SR-SEM to longitudinal SRM data. To date, there are only a few approaches that can be used to model longitudinal SRM data appropriately. In Nestler et al. (2017), for example, a combination of the univariate SRM with a linear multilevel growth model is proposed (called social relations growth model, SRGM). The model includes a (linear) time variable that predicts the repeated round-robin judgments of a single dyad (*i*, *j*). The intercept and the slope of this variable are assumed to contain a perceiver effect, a target effect, and a relationship effect; the SRGM thus assumes that the round-robin judgment of (*i*, *j*) changes as the perceiver effect of *i* changes, the target effect of *j* changes, and/or because the unique relationship effect changes. The SRGM is an interesting model, but its applicability is limited as it is tight to the linear growth model. This enabled the authors to calculate the covariance matrix of the round-robin data and to implement a corresponding estimation algorithm. However, this entails that it cannot, for example, be used to examine alternative growth curve specifications (e.g., quadratic or cubic trends) or autoregressive hypotheses concerning the SRM effects. Since the SR-SEM allows to define the mean and covariance structure of the round robin data in a very general way, one can very flexibly define different longitudinal models including different types of growth models or autoregressive path models. We therefore believe that the SR-SEM is an interesting approach to model longitudinal SRM data and future research should examine the performance of the SR-SEM in these contexts.

In our simulation study, the ML estimator performed well with regard to all three performance criteria. All simulation studies are limited, however, because they can only be used to investigate the statistical properties of an estimator within a specific range of simulation conditions. Hence, we believe that further simulation research is needed to examine the performance of our ML estimator with more complex models. It would be particularly interesting to conduct a simulation study to examine the ML estimator for models with low variance components or models in which some of the parameters of the person-level models, for example, are misspecified and to determine how this affects the parameter estimates of the other-level model. Finally, another objective for future research would be to compare our ML estimator with a Bayesian estimation approach. For example, it may be the case that the performance of the latter approach is better when the number of round-robin group members is smaller (see Lüdtke et al. 2013, 2018).

In sum, the present article introduced a combination of the SRM with the SEM framework, and we showed how the parameters of the model can be estimated with a ML approach. The illustration, the simulation, and the discussion hopefully showed that the suggested model is useful and that it offers numerous opportunities for future applied and methodological research.

## Appendix A

This Appendix contains another illustration of Eq. 6. Specifically, we use a single round-robin group (i.e., *G* = 1) with three round-robin group members (*n* = 3) that were asked to rate the other group members on two round-robin variables (i.e., *K* = 2) to present the model’s basic equation. In this case, the equation is

0.25 $$\begin{aligned} \varvec{y}&= \varvec{X} \varvec{\beta } + \varvec{Z}_1 \varvec{u}_1 + \varvec{Z}_2 \varvec{u}_2 + \varvec{Z}_3 \varvec{u}_3 + \varvec{W}_1 \varvec{r}_1 + \varvec{W}_2 \varvec{r}_2 + \varvec{W}_2 \varvec{r}_3 \Leftrightarrow \nonumber \\ \begin{pmatrix} y_{121} \\ y_{211} \\ y_{122} \\ y_{212} \\ y_{131} \\ y_{311} \\ y_{132} \\ y_{312} \\ y_{231} \\ y_{321} \\ y_{232} \\ y_{322} \end{pmatrix}&= \begin{pmatrix} 1 &{} 0 \\ 1 &{} 0 \\ 0 &{} 1 \\ 0 &{} 1 \\ 1 &{} 0 \\ 1 &{} 0 \\ 0 &{} 1 \\ 0 &{} 1 \\ 1 &{} 0 \\ 1 &{} 0 \\ 0 &{} 1 \\ 0 &{} 1 \end{pmatrix} \begin{pmatrix} \beta _1 \\ \beta _2 \end{pmatrix} + \begin{pmatrix} 1 &{} 0 &{} 0 &{} 0 \\ 0 &{} 0 &{} 1 &{} 0 \\ 0 &{} 1 &{} 0 &{} 0 \\ 0 &{} 0 &{} 0 &{} 1 \\ 1 &{} 0 &{} 0 &{} 0 \\ 0 &{} 0 &{} 1 &{} 0 \\ 0 &{} 1 &{} 0 &{} 0 \\ 0 &{} 0 &{} 0 &{} 1 \\ 0 &{} 0 &{} 0 &{} 0 \\ 0 &{} 0 &{} 0 &{} 0 \\ 0 &{} 0 &{} 0 &{} 0 \\ 0 &{} 0 &{} 0 &{} 0 \end{pmatrix} \begin{pmatrix} p_{11} \\ p_{12} \\ t_{11} \\ t_{12} \end{pmatrix} + \begin{pmatrix} 0 &{} 0 &{} 1 &{} 0 \\ 1 &{} 0 &{} 0 &{} 0 \\ 0 &{} 0 &{} 0 &{} 1 \\ 0 &{} 1 &{} 0 &{} 0 \\ 0 &{} 0 &{} 0 &{} 0 \\ 0 &{} 0 &{} 0 &{} 0 \\ 0 &{} 0 &{} 0 &{} 0 \\ 0 &{} 0 &{} 0 &{} 0 \\ 1 &{} 0 &{} 0 &{} 0 \\ 0 &{} 0 &{} 1 &{} 0 \\ 0 &{} 1 &{} 0 &{} 0 \\ 0 &{} 0 &{} 0 &{} 1 \end{pmatrix} \begin{pmatrix} p_{21} \\ p_{22} \\ t_{21} \\ t_{22} \end{pmatrix} + \begin{pmatrix} 0 &{} 0 &{} 0 &{} 0 \\ 0 &{} 0 &{} 0 &{} 0 \\ 0 &{} 0 &{} 0 &{} 0 \\ 0 &{} 0 &{} 0 &{} 0 \\ 0 &{} 0 &{} 1 &{} 0 \\ 1 &{} 0 &{} 0 &{} 0 \\ 0 &{} 0 &{} 0 &{} 1 \\ 0 &{} 1 &{} 0 &{} 0 \\ 0 &{} 0 &{} 1 &{} 0 \\ 1 &{} 0 &{} 0 &{} 0 \\ 0 &{} 0 &{} 0 &{} 1 \\ 0 &{} 1 &{} 0 &{} 0 \end{pmatrix} \begin{pmatrix} p_{31} \\ p_{32} \\ t_{31} \\ t_{32} \end{pmatrix} \nonumber \\&+ \begin{pmatrix} 1 &{} 0 &{} 0 &{} 0 \\ 0 &{} 1 &{} 0 &{} 0 \\ 0 &{} 0 &{} 1 &{} 0 \\ 0 &{} 0 &{} 0 &{} 1 \\ 0 &{} 0 &{} 0 &{} 0 \\ 0 &{} 0 &{} 0 &{} 0 \\ 0 &{} 0 &{} 0 &{} 0 \\ 0 &{} 0 &{} 0 &{} 0 \\ 0 &{} 0 &{} 0 &{} 0 \\ 0 &{} 0 &{} 0 &{} 0 \\ 0 &{} 0 &{} 0 &{} 0 \\ 0 &{} 0 &{} 0 &{} 0 \end{pmatrix} \begin{pmatrix} r_{121} \\ r_{211} \\ r_{122} \\ r_{212} \end{pmatrix} + \begin{pmatrix} 0 &{} 0 &{} 0 &{} 0 \\ 0 &{} 0 &{} 0 &{} 0 \\ 0 &{} 0 &{} 0 &{} 0 \\ 0 &{} 0 &{} 0 &{} 0 \\ 1 &{} 0 &{} 0 &{} 0 \\ 0 &{} 1 &{} 0 &{} 0 \\ 0 &{} 0 &{} 1 &{} 0 \\ 0 &{} 0 &{} 0 &{} 1 \\ 0 &{} 0 &{} 0 &{} 0 \\ 0 &{} 0 &{} 0 &{} 0 \\ 0 &{} 0 &{} 0 &{} 0 \\ 0 &{} 0 &{} 0 &{} 0 \end{pmatrix} \begin{pmatrix} r_{131} \\ r_{311} \\ r_{132} \\ r_{312} \end{pmatrix} + \begin{pmatrix} 0 &{} 0 &{} 0 &{} 0 \\ 0 &{} 0 &{} 0 &{} 0 \\ 0 &{} 0 &{} 0 &{} 0 \\ 0 &{} 0 &{} 0 &{} 0 \\ 0 &{} 0 &{} 0 &{} 0 \\ 0 &{} 0 &{} 0 &{} 0 \\ 0 &{} 0 &{} 0 &{} 0 \\ 0 &{} 0 &{} 0 &{} 0 \\ 1 &{} 0 &{} 0 &{} 0 \\ 0 &{} 1 &{} 0 &{} 0 \\ 0 &{} 0 &{} 1 &{} 0 \\ 0 &{} 0 &{} 0 &{} 1 \end{pmatrix} \begin{pmatrix} r_{231} \\ r_{321} \\ r_{232} \\ r_{322} \end{pmatrix} \end{aligned}$$

We explicitly note that a SRM cannot be fit to the data of a three-person round-robin group. We have included this example here only to present another illustration of the model.

## Appendix B

In this Appendix, we present the partial derivatives of $\varvec{{{\varSigma }}}_{\varvec{u}}$ and $\varvec{\mu }_{\varvec{u}_i}$ with respect to the elements in $\varvec{\varLambda }_{\varvec{u}}$, $\varvec{B}_{\varvec{u}}$, $\varvec{\varPhi }_{\varvec{u}}$, $\varvec{\varPsi }_{\varvec{u}}$, and $\varvec{{\varGamma }}_{\varvec{u}}$ (see also Mulaik 2009). We will not present the partial derivatives for $\varvec{{{\varSigma }}}_{\varvec{r}}$ and $\varvec{\mu }_{\varvec{r}_d}$ as they conform with the presented derivations.

The partial derivatives of $\varvec{{{\varSigma }}}_{\varvec{u}}$ with respect to the model matrices of the SR-SEM are:

0.26 $$\begin{aligned} \frac{\partial \varvec{{{\varSigma }}} _{\varvec{u} } }{\partial \lambda ^{u}_{ij}}&= 2 \cdot \varvec{1}_{ij} \varvec{\varPhi }_{ \varvec{u}} (\varvec{I} - \varvec{B}_{\varvec{u}})'^{-1} \varvec{\varLambda }_{\varvec{u}}' \nonumber \\ \frac{\partial \varvec{{{\varSigma }}} _{\varvec{u} } }{\partial b^{u}_{ij}}&= \varvec{\varLambda }_{\varvec{u}} \left[ (\varvec{I} - \varvec{B}_{\varvec{u}})^{-1} \varvec{1}_{ij} (\varvec{I} - \varvec{B}_{\varvec{u}})^{-1} \right] \varvec{\varPhi }_{ \varvec{u}} (\varvec{I} - \varvec{B}_{\varvec{u}})'^{-1} \varvec{\varLambda }_{\varvec{u}}' \nonumber \\&+ \varvec{\varLambda }_{\varvec{u}} (\varvec{I} - \varvec{B}_{\varvec{u}})^{-1} \varvec{\varPhi }_{ \varvec{u}} \left[ (\varvec{I} - \varvec{B}_{\varvec{u}})'^{-1} \varvec{1}_{ji} (\varvec{I} - \varvec{B}_{\varvec{u}})'^{-1} \right] \varvec{\varLambda }_{\varvec{u}}' \nonumber \\ \frac{\partial \varvec{{{\varSigma }}} _{\varvec{u} } }{\partial \phi ^{u}_{ij}}&= \varvec{\varLambda }_{\varvec{u}} (\varvec{I} - \varvec{B}_{\varvec{u}})^{-1} ( \varvec{1}_{ji} + \varvec{1}_{ij} - \varvec{1}_{ij} \cdot \varvec{1}_{ij}) (\varvec{I} - \varvec{B}_{\varvec{u}})'^{-1} \varvec{\varLambda }_{\varvec{u}}' \nonumber \\ \frac{\partial \varvec{{{\varSigma }}} _{\varvec{u} } }{\partial \psi ^{u}_{ij}}&= \varvec{1}_{ji} + \varvec{1}_{ij} - \varvec{1}_{ij} \cdot \varvec{1}_{ij} \nonumber \\ \frac{\partial \varvec{{{\varSigma }}} _{\varvec{u} } }{\partial \gamma ^{u}_{ij}}&= \varvec{0} \end{aligned}$$

where $\varvec{1}_{ij}$ is a matrix of respective size that contains a 1 at position (*i*, *j*) and zeros otherwise.

The partial derivatives of $\varvec{\mu }_{\varvec{u}_i}$ are

0.27 $$\begin{aligned}&\frac{\partial \varvec{\mu } _{\varvec{u} } }{\partial \lambda ^{u}_{ij}} = \varvec{1}_{ij} (\varvec{I} - \varvec{B}_{\varvec{u}})^{-1} \varvec{{\varGamma }}_{\varvec{u}}\varvec{x}_{\varvec{u}_i} \nonumber \\&\frac{\partial \varvec{\mu } _{\varvec{u} } }{\partial b^{u}_{ij}} = \varvec{\varLambda }_{\varvec{u}} \left[ (\varvec{I} - \varvec{B}_{\varvec{u}})^{-1} \varvec{1}_{ij} (\varvec{I} - \varvec{B}_{\varvec{u}})^{-1} \right] \varvec{{\varGamma }}_{\varvec{u}}\varvec{x}_{\varvec{u}_i} \nonumber \\&\frac{\partial \varvec{\mu } _{\varvec{u} } }{\partial \phi ^{u}_{ij}} = \varvec{0} \nonumber \\&\frac{\partial \varvec{\mu } _{\varvec{u} } }{\partial \psi ^{u}_{ij}} = \varvec{0} \nonumber \\&\frac{\partial \varvec{\mu } _{\varvec{u} } }{\partial \gamma ^{u}_{ij}} = \varvec{\varLambda }_{\varvec{u}} (\varvec{I} - \varvec{B}_{\varvec{u}})^{-1} \varvec{1}_{ij} \varvec{x}_{\varvec{u}_i} \nonumber \\&\frac{\partial \varvec{\mu } _{\varvec{y} } }{\partial \mu _{u,k}} . \end{aligned}$$

where $\varvec{1}_{ij}$ is a matrix of respective size that contains a 1 at position (*i*, *j*) and zeros otherwise, and $\varvec{1}_{k}$ is a column vector of respective size that contains a 1 at position *k* and zeros otherwise. Finally, we note that the partial derivative of $\varvec{\mu }_{\varvec{y}}$ with respect to $\beta$ is

0.28 $$\begin{aligned} \frac{\partial \varvec{\mu } _{\varvec{y} } }{\partial \beta _k} = \varvec{X} \varvec{1}_{k} \end{aligned}$$

where $\varvec{1}_{k}$ is again a column vector of respective size that contains a 1 at position *k* and zeros otherwise.

## Footnotes

1 If the data were collected in a balanced round-robin group design (i.e., the same number of round-robin group members were assessed in the *G* round-robin groups) and if no values are missing, then the dimension of $\varvec{Z}_i$ and $\varvec{W}_d$ would be ($K \cdot G \cdot n \cdot ( n - 1 ) )$ x ($2 \cdot K$) (here, *n* is the number of round-robin group members).

2 To this end, one has to write the self-ratings as round-robin variables and has to set certain constraints for the variance and covariance parameters of these variables. At present, this is a tedious procedure, but in the future, we intend to implement this in a more user-friendly way. We note that it is also possible to examine DIF for categorical moderators $\varvec{M}$ in the srm package. To do this, researchers have to define pseudo-items of the form $y \cdot m$, where *y* denotes the original variable and *x* denotes values of $\varvec{M}$.
