# Erratum to: Maximum Likelihood Estimation of a Social Relations Structural Equation Model

**DOI:** 10.1007/s11336-021-09793-y

**Published:** 2021-07-30

**Authors:** Steffen Nestler, Oliver Lüdtke, Alexander Robitzsch

**Affiliations:** 1 Institut für Psychologie, University of Münster, Fliednerstr. 21, 48149 Münster, Germany; 2 Leibniz Institute for Science and Mathematics Education, Kiel, Germany; 3 Centre for International Student Assessment, Kiel, Germany

## Erratum to: Psychometrika vol. 85, no. 4, 870–889 10.1007/s11336-020-09728-z

The article MAXIMUM LIKELIHOOD ESTIMATION OF A SOCIAL RELATIONS STRUCTURAL EQUATION MODEL, written by Steffen Nestler, was originally published electronically on the publisher’s internet portal on 22 January 2021 without open access. With the author(s)’ decision to opt for Open Choice, the copyright of the article changed on 12 July 2021 to $\copyright$ The Author(s) 2021 and the article is forthwith distributed under a Creative Commons Attribution.

